# The Assessment of Facial Expressions in Piglets Undergoing Tail Docking and Castration: Toward the Development of the Piglet Grimace Scale

**DOI:** 10.3389/fvets.2016.00100

**Published:** 2016-11-14

**Authors:** Pierpaolo Di Giminiani, Victoria L. M. H. Brierley, Annalisa Scollo, Flaviana Gottardo, Emma M. Malcolm, Sandra A. Edwards, Matthew C. Leach

**Affiliations:** ^1^School of Agriculture, Food and Rural Development, Newcastle University, Newcastle upon Tyne, UK; ^2^Department of Animal Medicine, Production and Health, University of Padova, Legnaro, Italy

**Keywords:** Piglet Grimace Scale, facial expression, tail docking, castration, pain

## Abstract

Many piglets are exposed to potentially painful husbandry procedures within the first week of life, including tail docking and castration, without the provision of either anesthesia or analgesia. The assessment methods used to evaluate pain experienced by piglets are often affected by low specificity and practical limitations, prompting the investigation of alternative methodologies. The assessment of changes in facial expression following a painful event has been successfully applied to several species. The objective of this pilot study was to evaluate the utility of a Grimace Scale applied to neonatal pigs to evaluate pain evoked by tail docking and castration. Eight female piglets, *Sus scrofa domesticus* (Landrace/Large White X synthetic sire line) underwent tail docking and 15 male piglets (75% Large White and 25% Belgian Landrace) were exposed to the castration procedure. Clear images of the faces of the piglets were collected immediately pre- and post-procedure. The images were used by experienced observers to identify facial action units (FAUs) which changed in individuals over this period, and a scoring scale was depicted in a training manual. A set of randomly selected images were then combined in a scorebook, which was evaluated after training by 30 scorers, blind to the treatment. The scale for most FAU was used with a high level of consistency across all observers. Tail docking induced a significant change (*P* < 0.05) in free moving piglets only in the “orbital tightening” FAU, whereas no change in any unit was observed in castrated piglets, which were restrained at the time of assessment. In this initial stage of development, orbital tightening seems to have the potential to be applied to investigate painful conditions in neonatal pigs. Nonetheless, more studies are needed to assess its full effectiveness and to evaluate the influence of possible confounds (e.g., handling stress) on the observed changes in FAUs.

## Introduction

Many of the current methods of assessing potentially painful conditions in farm animals are limited by their sensitivity and subjectivity. This, in turn, could mean that the alleviation of pain in these animals is inadequate. The main obstacle reported by farmers and veterinarians is the difficulty in recognizing and quantifying pain (1, 2), which consequently impedes the effective use of pain relief [e.g., Ref. (3)]. In order to obtain more quantifiable measures of pain, techniques such as the recording of changes in spontaneous behaviors (4, 5) or in evoked nociceptive responses [e.g., Ref. (6–8)] have been adopted in a range of species. Nevertheless, these techniques may not necessarily provide sensitive and reliable indicators of pain (9–12) as they often have practical constraints, such as the need for extensive observation bouts or complex experimental set-ups [e.g., Ref. (13)]. Pigs are frequently exposed as neonates to potentially painful conditions induced by husbandry procedures (e.g., tail docking, castration, ear-tagging/notching) (14). Due to the differences in pain perception associated with the early stages of maturation of the nervous system in humans (15, 16), it has been historically assumed that early-life injuries are not perceived as painful (17).

In contrast, previous reports suggest that tail docking and castration induce physiological and behavioral changes indicative of pain. Tail docking has been associated with changes in tail movements and position (18), body posture (19), vocalization (20), and neuroanatomical structures (21) considered to be indicative of pain. Similarly, castration has been linked to changes in behaviors (22, 23) and vocalization considered to be indicative of pain (24–26).

The complex nature of the pain experience, which encompasses physiological, molecular, and behavioral changes and is strongly influenced by individual variation, accentuates the need for a multifactorial approach, encompassing a variety of assessment methodologies. The analysis of facial expressions in animals represents a novel tool that has been shown to be informative in humans [e.g., Ref. (27)] and recently has been developed for rodents (28–30), rabbits (31), cats (32), horses (33), and sheep (34). These grimace scales have been shown to be an accurate and reliable method to identify post-procedural pain induced by a range of potentially painful procedures. Despite the underlying anatomical differences of the muscles of the face across species, some facial action units (FAUs) have been observed to consistently change in response to pain across species. These include FAUs related to the eyes, nose, cheeks, and mouth.

The aim of this study was, therefore, to explore the utility of using facial expressions in the study of pain arising from tail docking and castration in piglets. To achieve this, a set of FAUs were identified and submitted to 30 observers in order to evaluate their inclusion in the development of a Piglet Grimace Scale. The scores of the observers were correlated to their level of knowledge of pigs, to evaluate how familiarity with the animal species influenced the scores assigned to each image. Finally, we analyzed whether the changes in scores for each FAU were correlated to the changes in spontaneous behaviors that are considered to be indicative of pain in post-tail-docked piglets.

## Materials and Methods

### Tail Docking Study

#### Animals and Husbandry

All animal procedures were carried out under UK Home Office License (PPL 70/7919) and approved by the Animal Welfare Ethical Review Board of Newcastle University. Eight female piglets, *Sus scrofa domesticus* (Landrace/Large White X synthetic sire line) from the resident herd at Cockle Park Farm, Newcastle University, were used. All piglets were selected at random; however, any piglets with visible signs of injury, sickness, poor body condition, or abnormal behavior were not included in the selection. A total of four litters were enrolled in the study, and two piglets from each litter were used as experimental animals. The animals were 3 days old and, at the time of data collection, they had been exposed to teeth clipping as the only husbandry procedure within 24 h post-farrowing. The piglets were housed in farrowing pens measuring (1.8 m × 2.7 m) that consisted of a concrete and a partly slatted floor. Piglets had access to a creep area, which was heated by a 175 W infrared heat lamp (Interheat, Gyeonggi-do, South Korea) and had wood shavings as bedding material. Throughout the experiment, the room temperature ranged between 18 and 23°C with an 8/16 h light/dark cycle.

#### Tail Docking Procedure

Tail docking was carried out when the piglets were approximately 3 days of age. The procedure adhered to the regulation in force in the European Union, which permits tail docking within the first 7 days of age without the provision of anesthesia and analgesia (Council Directive 2008/120/EC). At the time of docking, each piglet was picked up and restrained by a trained operator through fixation of the fore and hind legs. A second observer recorded the total length at the lateral aspect of the tail (i.e., distance from the first proximal caudal vertebra to the tip) and drew a mark corresponding to one-third of the total length from the root of the tail. A gas-heated docking iron (East Riding Farm Services, Driffield, UK) was applied to the tail mark and the distal proportion of tail (i.e., remaining two-thirds of initial tail length) was removed. The animals were then placed into a filming pen (see details below) for 5 min before being returned to their home pen.

#### Collection of Images

**Figure 1:**
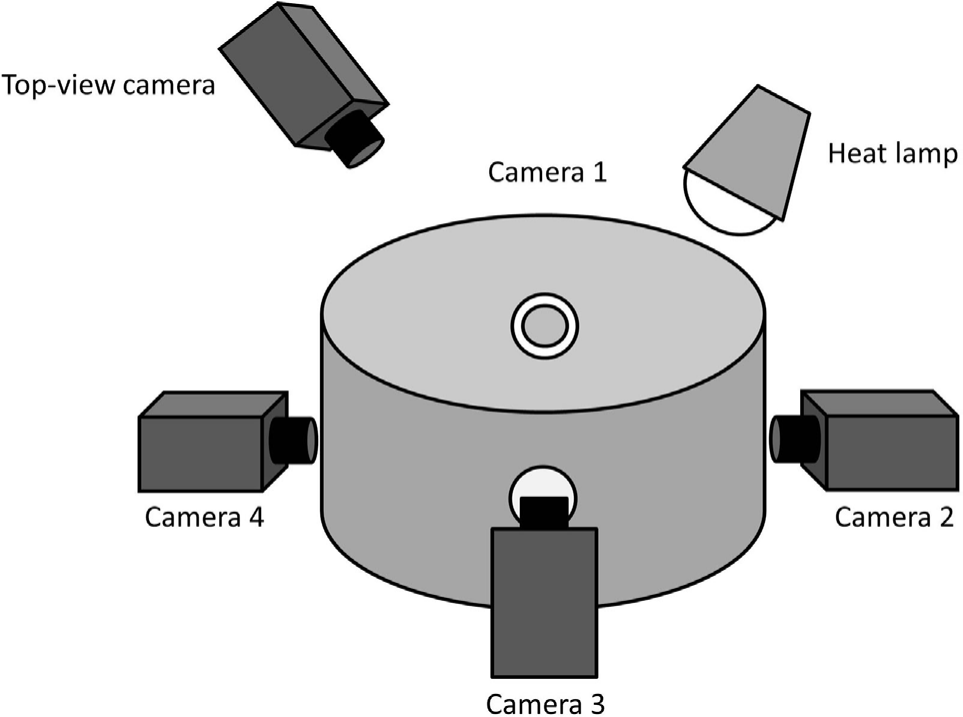
**Outline of the observation arena**. This indicates the position of the four cameras placed at equal distance to each other to record facial expressions of the piglets. The camera placed above the observation arena recorded the general activity of the piglets.

Video recordings of piglets were obtained pre- and post-docking by confining the animals in pairs in a custom-built observation arena (Figure 1) placed adjacent to the farrowing pen. The arena had a diameter of 56.5 cm and a height of 46 cm. It consisted of an opaque PVC wall, a rubber floor, with wood shavings provided as bedding, and an overhead IR heat lamp. Four holes/windows (5 cm diameter), equally distributed around the circumference of the arena, were made in order to allow recording with four high-definition video cameras (Canon Legria HF R606, Canon, Japan). The cameras were placed in correspondence with the holes/windows and the angles adjusted in order to obtain a complete view of the arena with the four combined recordings. Four tripods (Gorillapod Focus GP-8 Tripod, Joby, USA) were used to raise the cameras to a height of 19 cm. This set-up allowed clear video sequences of the piglets to be obtained as they were standing in the observation arena.

**Figure 2:**
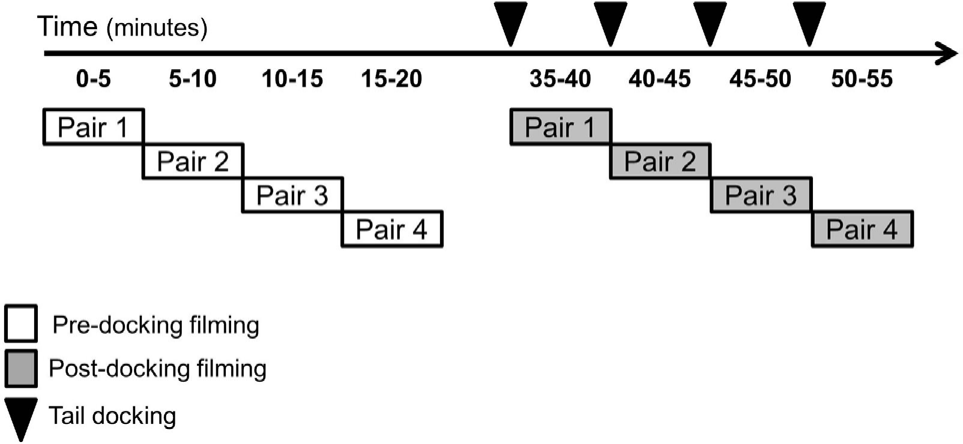
**Timeline of the experimental procedure**.

For pre-docking video recording, two experimental piglets (littermates) were placed in the arena and filmed for 5 min. Upon completion of pre-docking recording, the first pair was returned to the home pen and a second pair of undocked piglets was placed in the arena for filming. Once all four pairs had been recorded in the arena, post-docking filming began (Figure 2). Therefore, approximately 25–30 min following pre-docking filming, each piglet was tail docked and immediately placed in the arena with a companion piglet (randomly selected from the same litter). Piglets were always filmed in pairs to provide social contact and reduce distress associated with isolation from sow and littermates. Post-docking filming lasted 5 min. After each filming period, the piglets were returned to their home pen containing their littermates and mother. Pre- and post-docking, four still images for each piglet were obtained from each high-definition video sequence using VLC Media Player (VideoLAN, Paris, France). The images were extracted from videos by two observers who could not be blind to the treatment and who saved a frame whenever a clear, unobstructed and in-focus view of the entire face of the piglet was available on the screen.

#### Behavioral Observations

In addition to video recordings made at piglet head-height in order to obtain facial images, a top-view camera placed at a height of 1.2 m (Figure 1) allowed recording of the general behaviors of the two piglets pre- and post-tail docking. The videos were analyzed using CowLog 2.0 (35) following two behavioral sampling techniques. A focal continuous sampling was performed for 5 min pre- and 5 min immediately post-tail docking for each individual pig to record general indicators of locomotion and activity (Table 1). In addition, focal instantaneous scans were performed every 10 s to record the position of the tail (Table 1).

**Table 1:** **Description of behaviors and tail position recorded pre- and immediately post-docking**.

Behavior observed	Description
**Activity**	
Standing	Motionless, body weight supported by four legs
Walking	Slow movement one foot in front of the other
Sitting/kneeling	Motionless, body weight supported by hind quarters and front legs/by front carpal joints and hind legs
Lying	Motionless with shoulder or sternum in contact with the floor
**Interaction**	
Aggression	Forceful fighting, pushing with the head
Biting/chewing	Sharp short bite action toward other piglets/nibbling at littermates (ears, tail, foot)
**Tail position**	
High	Tail held above the level of the back, away from the body (including curled tail)
Middle	Tail held in a straight rigid position in line with the back and away from the body
Low	Tail relaxed, hanging below the level of the back
Tucked low	Tail is held tight against the body, covering the vulva and/or anus

*Adapted from Ref. (36)*.

### Castration

#### Animals and Husbandry

All animal procedures were approved after ethical review by Organismo preposto al benessere degli animali of the University of Padova (Prot. n. 19953 of the 26/03/2013). A total of 15 male piglets (75% Large White and 25% Belgian Landrace) were used in the study. All piglets were selected at random by a person not familiar with the nature of the study; however underweight, clinically sick, and cryptorchidic animals were excluded. A total of five litters were enrolled in the study and three piglets from each litter were used as experimental animals. The study was carried out in a commercial 400-sow farm located in the north-east of Italy. At the time of data collection, the piglets were 4 days old and had not been exposed to any previous husbandry procedure. They were housed with their dams in farrowing pens (1.5 m × 2.0 m) with fully slatted floors consisting of a wire mesh covered with rubber. Piglets were allowed access to a creep area heated by a 150 W radiant infrared heat lamp (Philips, Milan, Italy) with shredded paper as bedding material. The room temperature was maintained at 21 ± 2°C and the light/dark cycle was 8/16 h.

#### Castration Procedure

The piglets underwent routine surgical castration by a veterinarian at 4 days of age. The procedure adhered to the regulation in force in the European Union, which permits castration within the first 7 days of age without the provision of anesthesia and analgesia (Council Directive 2008/120/EC). Piglets were restrained between the legs of the veterinarian in a head down position exposing the genital area. After the scrotum was disinfected with a chlorhexidine-based antiseptic (Emulsan Suini, Tecnozoo, Piombino Dese, Italy), a skin incision of about 10 mm over each testicle was made with a scalpel. Testes were removed by cutting the spermatic cord and a local antibiotic treatment with chlortetracycline (Animedazon spray, Industria Chimica Fine, Palazzo Pignano, Italy) was then applied to both open wounds.

#### Collection of Images

Pre- and post-castration photographs were taken while the piglets were restrained as part of the surgical procedure. The veterinarian restrained the piglet by placing one hand under the thorax and held it so that the view of the face was not obstructed. A second operator took a minimum of five clear photographs with a high-definition camera (Digital Ixus 60, Canon, Japan) placed at approximately 50 cm away from the face of the piglet. Collection of images took 90 s, immediately before and after castration.

### Image Selection and Processing

Images were considered acceptable when the entire face of the animal was contained in the frame with all target facial regions in focus. Images of piglets with their eyes closed or otherwise covered by the ears were discarded. Each image was cropped by a non-participating operator who could not be blind to the treatment in order to make only the head of the animal visible and to guarantee blinding by not revealing the rest of the body of the piglet, in accordance with the method set out by Langford et al. (28). For the tail-docked piglets, four pre- and four post-docking images per piglet were selected for the analysis. For the castrated piglets, one pre- and one post-surgery image per piglet were selected for the analysis. These images were randomly selected from a larger pool of images of each animal at each time point (i.e., pre- and post-procedure), using a random number generator to avoid observer bias.

### Development of the Facial Expression Scale

**Figure 3:**
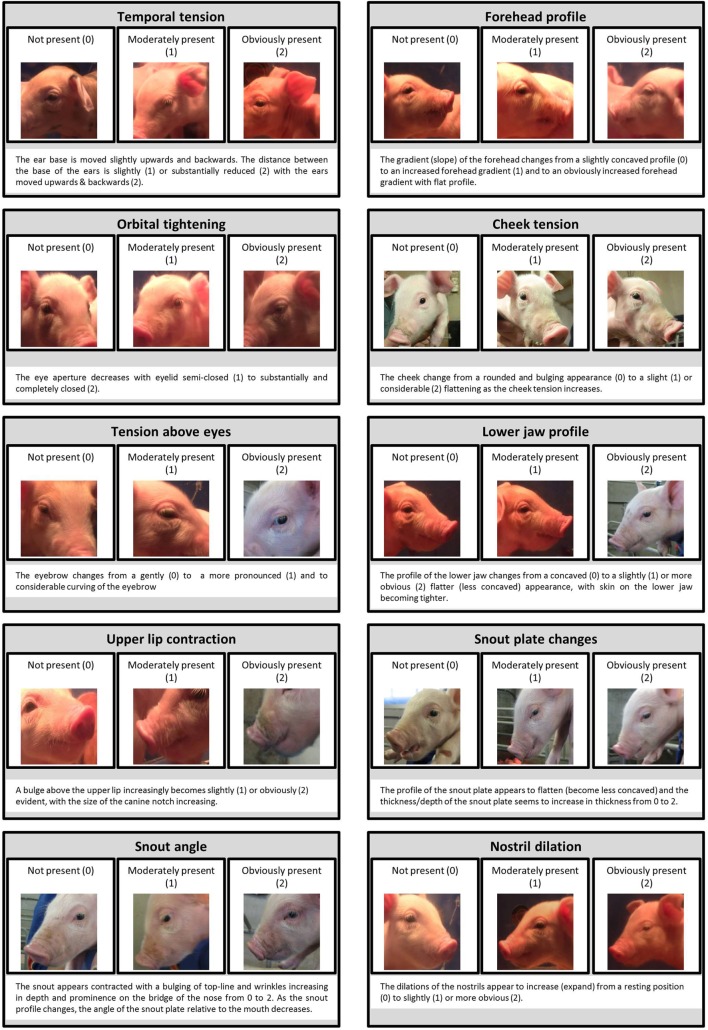
**Collection of images and explanations for each of the 10 facial action units (FAUs) included in the development of the Piglet Grimace Scale**. For each FAU, an explanation of the 3-point scale is included (0 = not present, 1 = moderately present, 2 = obviously present).

The selected images from both studies were combined into a collage comprising a total of 94 individual images, divided into 47 pre- and 47 post-procedure photographs. A team of experienced researchers from Newcastle University and the University of Padova, who were blind to both the treatment and the time point, independently compared the images from pre and post-procedure within each individual to identify the facial features that changed. Those features that consistently changed (i.e., in over 50% of the animals) were used to generate a proto Piglet Grimace Scale that comprised 10 potential facial features or FAUs. The FAUs included: temporal tension, forehead profile, orbital tightening, tension above the eyes, cheek tension, snout angle, snout plate change, upper lip contraction, lower jaw profile, and nostril dilation (Figure 3). This proto-scale was then used by 30 treatment and time-point blind participants to score the 94 images. The images were presented in a randomized order to each participant. The participants were asked to familiarize themselves with the FAUs before scoring the images, using a detailed manual that provided guidelines on how to assign a score for each FAU based on a 4-point system [0 = not present, 1 = moderately present, 2 = obviously present, 9 = don’t know, e.g., Ref. (28)]. The participants were selected from different professional backgrounds and their degree of knowledge of pigs ranged from 0 (“No knowledge”) to 5 (“Expert”) (Average = 1.6).

### Statistical Analysis

All statistical analyses were performed with SPSS version 22.0 for Windows (SPSS Inc., Chicago, IL, USA). Differences were considered to be statistically significant if *P* < 0.05. Median values of the scores given by each observer to the images and for each individual FAU were calculated. A non- parametric Wilcoxon matched pairs test was used to detect differences between pre- and post-procedure scores for each individual FAU for tail docking and castration, separately. In order to evaluate the level of agreement among observers, an Inter-class correlation coefficient (ICC) was calculated for the scores of each FAU. The correlation between the level of knowledge of the scorers and the median scores assigned to each image was analyzed with a Spearman rank correlation. A paired *t*-test was performed to analyze pre- to post-tail docking changes for each behavioral variable and tail position. A Spearman correlation coefficient analysis was then used to correlate the difference in the duration of each behavioral variable (pre- vs. post-tail docking) with the difference of the median scores for any action unit that was shown to change significantly from pre- to post-procedure.

## Results

**Figure 4:**
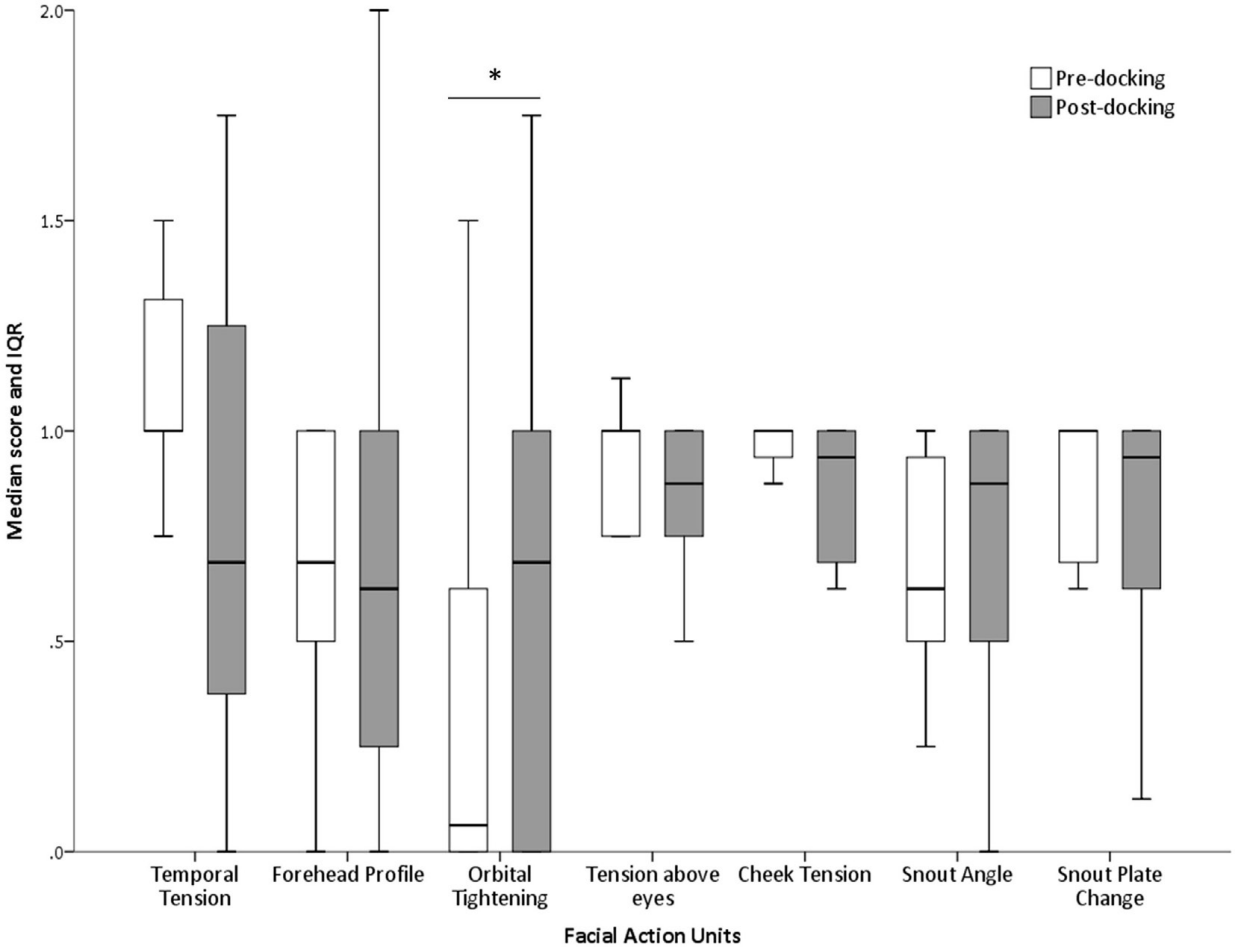
**Changes in FAU scores following tail docking**. Median (+IQR) of all scores assigned to each FAU pre and post-procedure. The asterisk denotes significant difference (*P* < 0.05).

The proportions of “don’t know” (i.e., unable to score) responses varied substantially between FAUs, from 2% for the “orbital tightening” to 72% for the “nostril dilation” (Table 2). The average number of “don’t know” responses given by each scorer to each image and for each FAU is also reported in Table 2. “Upper lip contraction,” “nostril dilation,” and “lower jaw profile” were excluded from further analysis as, for over 30% of the images, the participants could not consistently see these FAUs. The refined scale exhibited high inter-observer reliability with an overall ICC of 0.97. Similarly, high ICC values were recorded for each of the individual action units: temporal tension 0.97, forehead profile 0.82, orbital tightening 0.95, tension above the eyes 0.96, cheek tension 0.86, snout angle 0.90, and snout plate change 0.92. No correlation between the level of pig knowledge of the observers and their scores was observed (temporal tension: *r*_s_ = −0.277, *P* = 0.139; forehead profile: *r*_s_ = −0.088, *P* = 0.656; orbital tightening: *r*_s_ = −0.193, *P* = 0.307; tension above the eyes: *r*_s_ = −0.040, *P* = 0.833; cheek tension: *r*_s_ = −0.210, *P* = 0.266; snout angle: *r*_s_ = −0.268, *P* = 0.152; snout plate change: *r*_s_ = −0.057, *P* = 0.766). “Orbital tightening” was the only FAU that significantly changed from pre- to post-tail docking (temporal tension: *Z* = −1.276, *P* = 0.202; forehead profile: *Z* = −0.000, *P* = 1.000; orbital tightening: *Z* = −2.041; *P* = 0.041; tension above the eyes: *Z* = −0.271, *P* = 0.786; cheek tension: *Z* = −0.962, *P* = 0.336; snout angle: *Z* = −0.535, *P* = 0.593; snout plate change: *Z* = −1.134, *P* = 0.257), with post-docking images showing significantly higher median scores than pre-docking (Figure 4). No change in median scores of any of the FAUs was observed in association with castration (temporal tension: *Z* = 0.000, *P* = 1.000; forehead profile: *Z* = −1.080, *P* = 0.280; orbital tightening: *Z* = −1.634; *P* = 0.102; tension above the eyes: *Z* = −1.006, *P* = 0.314; cheek tension: *Z* = −0.877, *P* = 0.381; snout angle: *Z* = −1.576, *P* = 0.115; snout plate change: *Z* = −0.831, *P* = 0.406).

**Table 2:** **Percentages and average number per scorer of “don’t know” responses for each FAU based on a total of 94 images**.

Facial action unit	Unable to score (%)	Average per scorer
Temporal tension	4	3
Forehead profile	17	11
Orbital tightening	2	1
Tension above the eyes	5	3
Cheek tension	11	7
Upper lip contraction	36	21
Lower jaw profile	46	28
Snout angle	18	10
Snout plate changes	27	16
Nostril dilation	72	44

**Figure 5:**
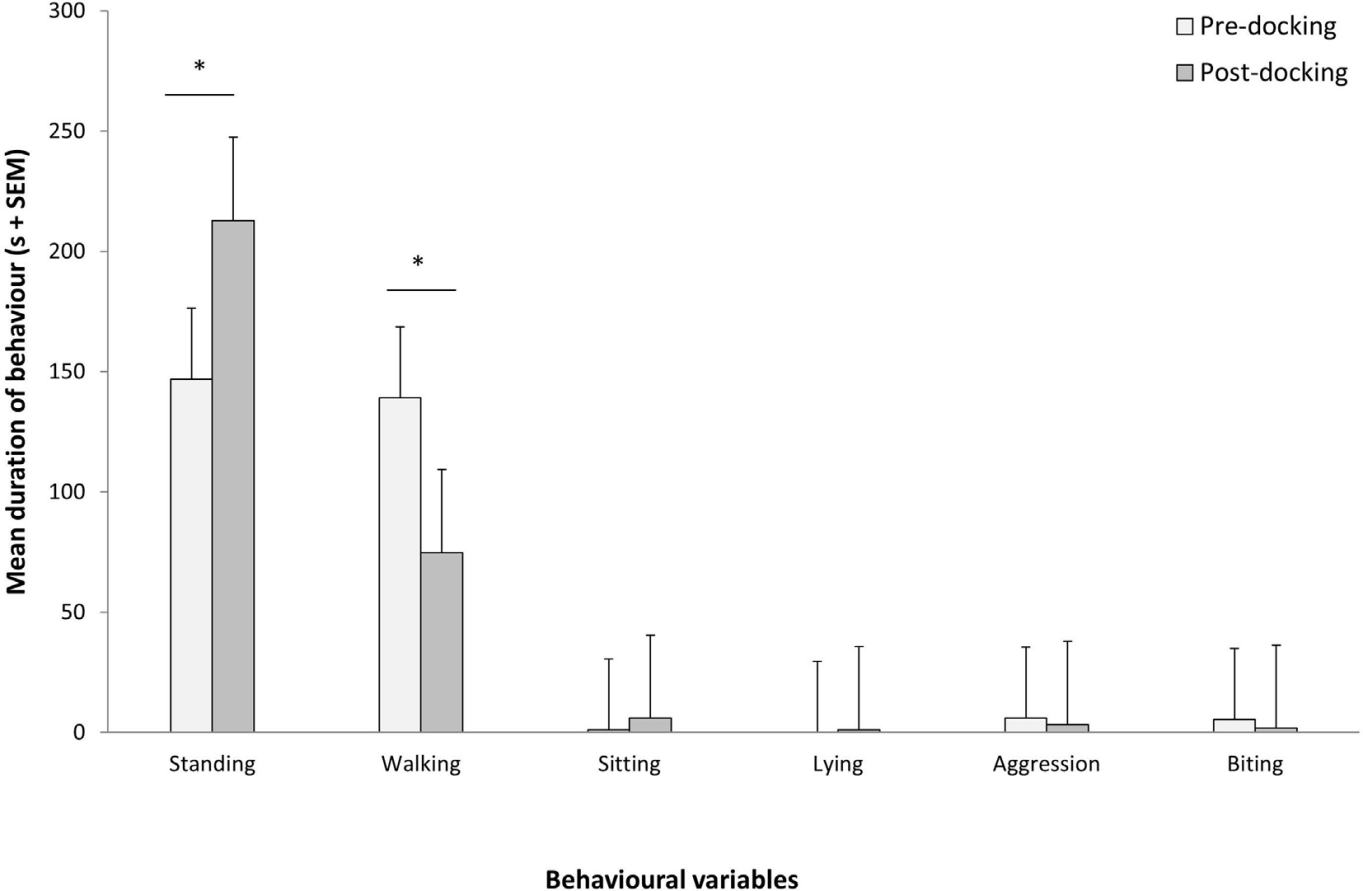
**Changes in pre vs. post-tail docking behaviors**. Mean (+SEM) duration of each behavior recorded for 5 min pre and immediately following tail docking. The asterisk denotes significant difference (*P* < 0.05).

**Figure 6:**
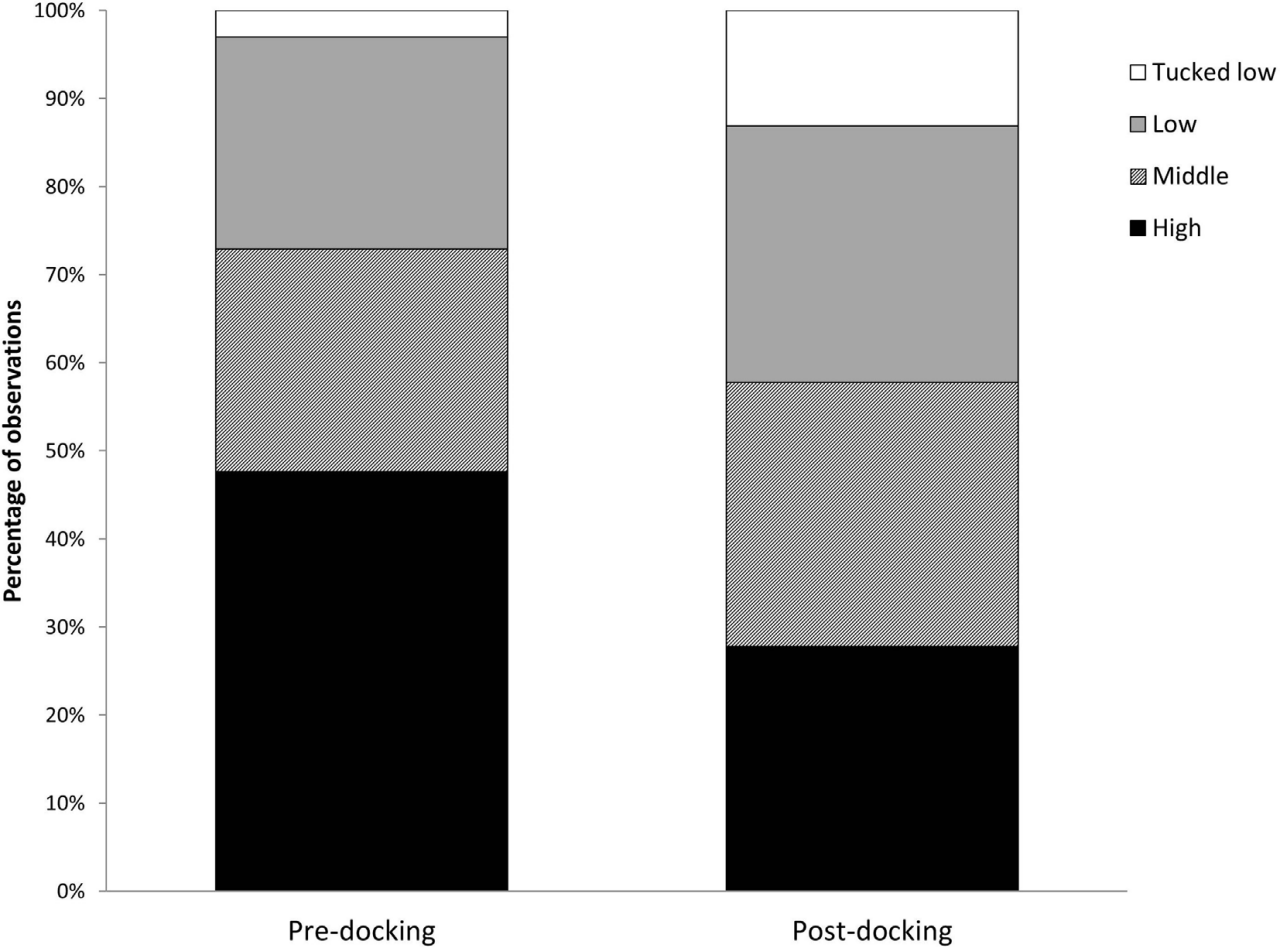
**Changes in tail posture**. Percentage of total observations of tail posture carried out pre and immediately post-tail docking.

Piglets undergoing tail docking spent a significantly greater amount of time standing in the 5-min post-procedural observation period (pre: 147 s vs. post: 213 s, *t* = −4.196, *P* = 0.004) (Figure 5). The time spent walking significantly decreased (pre: 139 s vs. post: 75 s, *t* = 4.415, *P* = 0.003). The other behavioral variables were not significantly different between pre- and post-tail docking (sitting/kneeling: *t* = −0.851, *P* = 0.423; lying: *t* = −1.155, *P* = 0.286; aggression: *t* = 0.857, *P* = 0.420; biting/chewing: *t* = 1.117, *P* = 0.301). The prevalence of the four identified tail postures did not change significantly from pre- to post-tail docking; however, there was a tendency (*t* = 1.861, *P* = 0.100) for the percentage of “tail high” position to be reduced from pre- to post-docking (Figure 6). There was no correlation between the difference in the duration of each behavioral variable (pre- vs. post-tail docking) and the difference of the median scores of “orbital tightening” (standing: *r*_s_ = 0.329, *P* = 0.426; walking: *r*_s_ = 0.222, *P* = 0.597).

## Discussion

To the best of our knowledge, this is the first paper on the analysis of changes in the facial expressions of piglets in relation to potential pain arising from tail docking or castration. The seven FAUs finally included in this preliminary scale were scored with high consistency by 30 observers. Despite this level of consistency, “orbital tightening” was the only FAU to significantly change from pre- to post-tail docking procedure, with no detectable changes in piglets undergoing castration. This change in orbital tightening, along with changes in spontaneous behaviors and the changes in tail posture seem to provide evidence supporting the occurrence of acute pain following tail docking.

Interestingly, the change in “orbital tightening” is observed in all existing grimace scales developed for other animal species (28, 29, 31, 33, 34). In order to develop a scale specific to piglets, several FAUs were identified by a team of experienced animal scientists and veterinarians. Some of the FAUs corresponded to those reported in other species (e.g., temporal tension, orbital tightening), whereas new FAUs were introduced (e.g., snout angle and snout plate changes) as they were highly specific to the facial anatomical features of the pig. In particular, we included several FAUs associated with the snout because of its complex morphology resulting from its multiple functions (e.g., rooting in the soil, manipulation) (37). The occurrence of a change in orbital tightening as well as the absence of significant changes in other FAUs should be interpreted with particular caution. The confining of piglets in pair (tail docking) or alone (castration) in a novel environment away from the sow and littermates may have induced stress in these animals and so influenced the exhibition of facial expressions. Pigs are a highly social species, particularly reactive to stressful conditions, and the fear responses become increasingly evident in younger animals (38). The presence of fear or anxiety may have influenced or concealed changes in FAUs, since many of the action units that comprise facial expressions may not be context-specific and so may correspond to different emotional states (other than pain), as observed in humans (39). In the case of castration, the physical restraint of the piglet while images were taken is likely to have induced even greater stress. From this study, we are, therefore, unable to differentiate between the potential effects of pain and stress on facial expressions in either the pre- or post-procedure period. The influence of fear and anxiety on facial grimacing has been reported in rodents (40, 41), suggesting that the observed changes may be sensitive to more than one emotional state or they may be linked to the emotional arousal, commonly observed in animals experiencing fear, anxiety, stress, and pain. Further studies are, therefore, required in piglets to differentiate the effects of pain from other emotional states which might be induced by procedures during the assessment process.

In the current tail docking study, we attempted to reduce procedural stress by recording the animals in pairs and by placing the observation arena at a very short distance from the sow, therefore offering auditory and olfactory cues. Despite the possibility of stress due to novelty and temporary removal from the sow affecting pain-related facial expressions of tail-docked piglets, a change in “orbital tightening” was detected. This was in contrast with the absence of changes in scores of castrated piglets. The different experimental approach, which involved handling and restraint of the piglets while being photographed, does not allow unambiguous interpretation on the feasibility of the method for detecting castration pain.

Compared to previously reported grimace scales, this is the first developmental work performed on non-human neonates. The likely rapid morphological changes experienced by pigs in this phase of their life has the potential of resulting in high levels of individual variation in some of the facial features included in this study (42). In order to evaluate whether the changes in facial expressions are purely pain-specific, future studies should focus on the reversible effect of analgesia or the preventive use of local anesthesia on the post-procedural changes in FAUs, incorporating the principle and criteria suggested by Sneddon et al. (12).

The overall inter-observer reliability of 0.97 reported here is greater than that reported for other grimace scales [mouse: 0.90 (28); rat: 0.90 (29); rabbit: 0.91 (31); horse: 0.92 (33); sheep: 0.86 (34)]. This finding, combined with the lack of a correlation between the level of knowledge of the observers and the median scores assigned to each image, suggests that the instructions included in the manual provided sufficient training and the human observers are able to consistently score facial expressions in this non-human species.

The difficulty experienced by the observers in assigning a score to “upper lip contraction,” “lower jaw profile,” and “nostril dilation” may have been caused by a suboptimal quality of the images. In particular, the light source provided by a lamp suspended over the observation arena may have caused unwanted shadowing of the ventral aspect of the face of the piglets. In addition, the rapid, jerky-like movements of piglets at this particular age may have interfered with the automatic focus adjustment of the video cameras, therefore recording a higher proportion of unclear images. In sheep, the lip, jaw profile, nostril, and philtrum positioning were also characterized by a lower degree of reliability in contrast to a greater consistency associated with the orbital area and ear position (34). An analogous situation had been previously reported in horses (33). In both reports, photographs taken at less effective angles were indicated as the main source of negative impacts on effective scoring. Although the set-up described in this study allowed the collection of images at more effective angles, providing a more uniform source of light would be strongly recommended for future applications in an attempt to obtain images of acceptable standard quality and potentially avoiding the exclusion of photographs due to poor light.

The observed changes in locomotion of the piglets immediately following tail docking may be interpreted as a reflection of the pain induced by the procedure. The significant reduction in time spent walking and the increase in standing, could be justified by the attempt made by the piglet to avoid unnecessary physical stimulation of the tail, such as would be evoked by touching the rear end of the body against the walls of the observation arena. Sensitization of the mechanical nociceptive fibers in the tail can be expected as a result of an injury of this nature, which is similar to the process caused by skin and muscle incisions in piglets (43). While locomotory changes have been reported following castration (25), previous reports on spontaneous behaviors immediately following tail docking have not indicated such changes, with similar durations of standing and walking performed by the piglets pre- vs. post-procedure (19, 44). Nonetheless, previous behavioral data were collected while the piglets were housed in their litters in home pen conditions. Therefore, the different experimental set-up reported here should be taken into account. The introduction to a novel environment may have been expected to cause an increase in exploratory behavior, potentially leading to longer time spent walking. Alternatively, more walking may have been expected as the piglets attempted to escape the arena. The observed difference is unlikely to be a consequence of habituation to the test arena as, in a previous study, pigs exposed to an open arena twice over two consecutive days were reported to spend the same amount of time standing (45). The change observed in this study could be the result of acute post-tail docking pain; however, due to the small sample size, more investigation on the efficacy of these specific indicators is required.

Immediately following tail docking, we observed a change in tail posture, with an increase in “middle,” “low,” and “tucked low” and a decrease in the “high” tail position. Although not statistically significant, these results seem to be in agreement with previous observations of increased “tail jamming” behavior (18, 44). The sensitization experienced locally in the tail may have induced this posture, which could be interpreted as a protective behavior to avoid stimuli from the environment (i.e., tail touching the walls of the arena or the other piglet). Since tucking the tail has not been observed solely in response to tail docking, but also in response to tail biting in later life (46), more research should be carried out to assess its value as a post-procedural pain indicator.

## Conclusion

Following this pilot study, the Piglet Grimace Scale requires considerable further development as a potential tool to detect post-procedural pain in neonatal pigs. The absence of a detected change in expression following castration highlights the possible confounds with other stressors associated with the procedure during measurement, and further studies should be carried out that avoid the limitations highlighted thus far. In addition, changes associated with other husbandry procedures that are known for evoking detectable signs of pain in piglets should be carried out. Furthermore, the evaluation of the efficacy of local anesthetic or analgesic treatments would help understand whether the observed changes in facial expressions are reversible, therefore supporting their use as behavioral indicators of ongoing pain. In addition, future investigations could aim at exploring the changes in the FAUs reported in this study in a different context, such as for example in the presence of a controlled stressor. A full characterization of the Piglet Grimace Scale would give the opportunity to implement it as a cost-effective tool for the on-farm assessment of painful and or distressing conditions induced by husbandry in piglets. Finally, it would provide the basis for the development of a scale specific to non-neonatal pigs, which would benefit the assessment of pain in relation to spontaneous health conditions (e.g., lameness induced by degenerative joint diseases or infection) that are normally observed in older animals.

## Author Contributions

PG designed the study, collected and analyzed the data for this report, and wrote the manuscript for submission. ML designed and analyzed the data and contributed to the preparation of the manuscript. SE participated in the design of the study, the data analysis, and the writing of the manuscript. AS, FG, EM, and VB participated in the design of the study, performed the data collection and analysis, and commented on the manuscript. All the authors approved the final manuscript.

## Conflict of Interest Statement

The authors declare that the research was conducted in the absence of any commercial or financial relationships that could be construed as a potential conflict of interest.
